# Squamous Cell Carcinoma of the Vulva in Geriatric With Clinical Features of Papillomatous Plaque Resembling Anogenital Warts Caused by Human Papillomavirus Types 6 and 11

**DOI:** 10.1155/crdm/1730469

**Published:** 2025-08-27

**Authors:** Pati Aji Achdiat, Fathia Rianty, Risa Miliawati Nurul Hidayah, Miranti Pangastuti, Hermin Aminah Usman, Ida Ayu Made Niki Putri Ashrita, Retno Hesty Maharani

**Affiliations:** ^1^Department of Dermatology and Venereology, Faculty of Medicine, Universitas Padjadjaran, Bandung, West Java, Indonesia; ^2^Department of Anatomical Pathology, Faculty of Medicine, Universitas Padjadjaran, Bandung, West Java, Indonesia

**Keywords:** anogenital warts, low-risk human papillomavirus (HPV), sexually transmitted, squamous cell carcinoma (SCC)

## Abstract

Squamous cell carcinoma (SCC) of the vulva is caused by disturbances in the proliferation and differentiation of the squamous epithelium, which can be associated with human papillomavirus (HPV) infection. Vulvar SCC is usually caused by high-risk HPV types, although there are some cases that are caused by low-risk HPV types. The clinical manifestations of vulvar SCC vary, one of which can resemble anogenital warts. A case of vulvar SCC was reported in a 75-year-old woman with a lesion that appeared as a yellowish-white papillomatous plaque on the vulva. From the history taking, it was found that the skin disorder had a chronic course. A skin biopsy was performed on the patient and from histopathological examination found hyperplastic, parakeratotic, and acanthotic stratified squamous epithelial cells with koilocytosis. In addition, there was evidence of tumor cell invasion in the connective tissue stroma with polymorphic and hyperchromatic nuclei, supporting a diagnosis of well-differentiated vulvar SCC. Genotyping polymerase chain reaction revealed the presence of HPV types 6 and 11 deoxyribonucleic acid. The risk of malignancy in low-risk HPV must be considered, especially in the elderly. HPV-dependent SCC caused by low-risk HPV types 6 and 11, though uncommon but still possible, with clinical manifestations that resemble anogenital warts.

## 1. Introduction

Squamous cell carcinoma (SCC) is a malignancy that originates from squamous epithelial cells [1, 2]. SCC generally appears on parts of the body exposed to sunlight but can also occur on the vulva [1, 3]. The incidence of vulvar SCC is approximately 1–4 per 100,000 women, particularly in older women [4]. Risk factors for vulvar SCC, in addition to old age [1, 3], include immunodeficiency conditions, smoking habits [4], alcohol consumption [5], previous chronic inflammation of the vulva, and human papillomavirus (HPV) infection [6, 7]. Importantly, the incidence of SCC varies significantly across regions, with the highest age-standardized prevalence rates observed in high-income countries such as the United States and Australia, and markedly lower rates in low-SDI regions like Western Sub-Saharan Africa, according to the Global Burden of Disease 2021 study [8]. HPV belongs to the *Papillomaviridae* family, which has double-stranded deoxyribonucleic acid (DNA) [9]. HPV has two types of virus groups [1], low-risk types which generally cause benign lesions such as anogenital warts, and high-risk types, which generally cause malignant lesions [1, 10]. However, reports have linked low-risk HPV infections to several cases of genital malignancies, including vulvar SCC [5, 9, 11, 12]. Clinical manifestations of vulvar SCC can include thickening or discoloration of the skin, with lesions such as macules, plaques, ulcers [5], or tumors [3, 5]. The histopathological features of vulvar SCC generally resemble the condyloma acuminatum type of anogenital warts, characterized by atypical cells and koilocytosis [1]. However, in vulvar SCC, there are also findings of the epidermis with numerous mitotic cells, loss of cell maturation, and cells with hyperchromatic nuclei [5, 13, 14]. The epidermis is usually thickened and shows features of papillomatosis [13], acanthosis, hyperkeratosis, and parakeratosis [5]. Management of vulvar SCC includes surgery, chemotherapy, radiation therapy, and counseling [15]. The choice of therapy for vulvar SCC is determined based on the clinical stage and the patient's condition [16, 17]. This case report presents a rare case of vulvar SCC in a 75-year-old woman, characterized by a clinical manifestation of papillomatous plaque and confirmed by polymerase chain reaction (PCR) genotyping to be positive for low-risk HPV types 6 and 11.

## 2. Case Report

**Figure 1:**
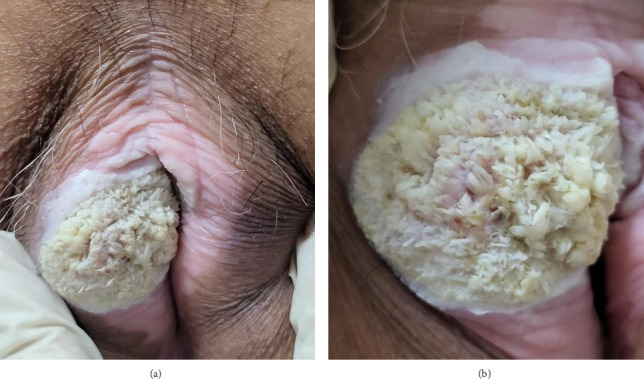
(a-b) White-to-yellowish genital lesion.

**Figure 2:**
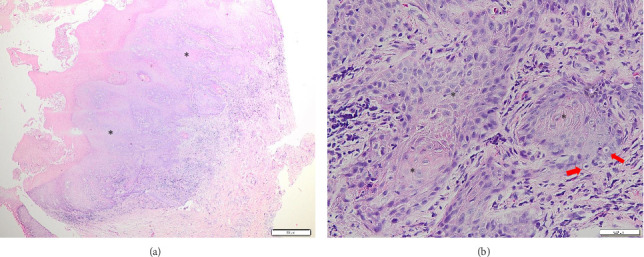
(a-b) Histopathological examination results. (a) The epithelial cells that appear to have transformed into a tumor mass (black asterisks) consisting of round to polygonal cells with invasion into the underlying connective tissue stroma, which consists of round to oval cells that are hyperplastic, dense, and clustered. (b) Koilocytosis is observed in some nuclei (red arrows), indicating HPV-associated cytopathic changes.

A 75-year-old woman presented with complaints of a yellowish-white lump in the genital area which was sometimes itchy and painful. The first complaint was noticed 6 months prior to seeking medical attention in the form of a skin-colored papule that was neither itchy nor painful. Approximately 1 month later, the skin-colored papules increased in number and size and became itchy. Three months prior to seeking treatment, the previously existing papules changed color to yellowish-white, increased in size, and coalesced into a plaque. The patient visited a clinic and was treated with topical bufacort and oral medications, but the symptoms did not improve. Two weeks later, the skin lesion grew to the size of a ping pong ball and became increasingly itchy and painful, especially when rubbed. The complaint about skin lesions becoming fragile and easily bleeding is denied. The patient sought treatment at Santo Jusuf Hospital and was then referred to Dr. Hasan Sadikin Hospital (RSHS) for further evaluation. There was no history of other sexually transmitted infections (STIs). The patient had coitarche with her husband at the age of 23, always with genital–genital intercourse, and had never used a condom. Neither the patient nor her husband had a history of promiscuity. Neither the patient nor her husband had received HPV vaccination. On physical examination, vital signs were within normal limits, the patient was normoweight, and no lymph node enlargement was found. Venereological examination revealed a solitary lesion on the vulva, round in shape, 3.6 cm in diameter and 0.3-cm thick, well demarcated, raised, dry, and yellowish-white in color, with a papillomatous surface (Figures 1(a) and 1(b)). Routine hematologic and serologic tests for syphilis, hepatitis B, and human immunodeficiency virus (HIV) were within normal limits. A punch biopsy was performed from the skin lesion for histopathological examination, which revealed hyperplastic stratified squamous epithelium with parakeratosis, acanthosis, and some nuclei showing koilocytosis. The epithelial cells had transformed into a tumor mass composed of polygonal round cells with tumor cell invasion into the underlying connective tissue stroma composed of round, oval cells growing hyperplastically, densely packed, and clustered. The nuclei were polymorphic and hyperchromatic (Figures 2(a) and 2(b)). The histopathological examination supported the diagnosis of well-differentiated SCC. HPV DNA detection was performed using conventional PCR targeting the L1 region of the viral genome. Specific primers for HPV types 6, 11, 16, and 18 were used. Positive and negative controls were included in each PCR run to ensure the accuracy and reliability of the amplification process. The positive control consisted of known HPV-positive DNA samples, while nuclease-free water was used as a negative control. PCR for HPV from the lesion revealed positive results for HPV types 6 and 11 and negative results for HPV types 16 and 18. The final diagnosis for the patient was well-differentiated SCC of the vulva. The patient was referred to the Gynecologic Oncology division for further evaluation. She underwent additional workup including pelvic ultrasound, Pap smear, and bilateral inguinal fine-needle aspiration biopsy (FNAB), which revealed no evidence of lymph node involvement or distant metastasis. Surgical excision of the vulvar lesion was subsequently performed. However, the patient chose to continue her postoperative care at another hospital and was thus lost to follow-up from our center.

## 3. Discussion

SCC is a type of malignancy that occurs in organs lined with squamous epithelium and is the second most common cause of skin cancer in the United States, with an incidence of over one million cases per year [17]. SCC usually occurs on areas of the body exposed to sunlight [2, 18] but can also occur in the genital area, such as the vulva and vagina [1, 3]. The prevalence of vulvar SCC ranges from 3% to 5% of all genital malignancies in women and is the most common type of tumor occurring in the vulva and vagina [3, 9, 16]. According to a study conducted by Ouh et al. [19] between 2014 and 2018, the prevalence of vulvar cancer is higher in women over 40 years of age than in women under 40 years of age, with the highest prevalence occurring in women over 70 years of age.

Vulvar SCC can be divided into two types based on its etiopathogenesis, HPV-dependent and HPV-independent types [4, 5, 9, 13]. Vulvar SCC of the HPV-independent type commonly occurs in women who experience chronic inflammation such as lichen sclerosus and lichen planus [4, 5]. Meanwhile, vulvar SCC of the HPV-dependent type often occurs in women aged 35–65 years, with risk factors influencing the development of HPV-dependent vulvar SCC including history of genital warts or previous sexually transmitted diseases, multiple sexual partners, immunosuppression [5, 11], low socioeconomic status, and alcohol consumption [1, 5, 20]. The precursor lesion for HPV-dependent vulvar SCC is called high-grade squamous intraepithelial lesion (HSIL) [9, 21], characterized by the presence of atypical cells [1, 5, 13]. HPV-related vulvar SCC is usually caused by high-risk HPV types such as types 16, 18, 31, 33, and 45 [5, 21], although in some cases, it can be caused by low-risk HPV types such as types 6 and 11 [4, 5, 22]. Although its association with vulvar cancer has not yet been explained, there are reports showing that HPV-6 and HPV-11 can cause malignant lesions in noncervical locations. Low-risk HPV has been reported in up to 5.5% of penile cancers and 87.5% of laryngeal cancers. Laryngeal lesions associated with HPV-11 are thought to be more aggressive than HPV-6 lesions [23].

Low-risk HPV tends to cause benign warts and anogenital lesions, while high-risk HPV tends to cause cancer and malignant lesions [5, 9, 11, 12]. High-risk HPV types, such as types 16 and 18, bind to p53 and Rb proteins up to 10 times stronger than low-risk HPV types [24, 25]. The mechanism by which low-risk HPV causes vulvar SCC remains unclear [5]. The patient in this case report has HPV-dependent vulvar SCC, as the PCR genotyping test results were positive for HPV types 6 and 11 from the skin tissue biopsy specimen.

Cancer can be caused by various factors. Most malignancies in adults show an increase in incidence with age [26]. Vulvar SCC generally occurs in postmenopausal women, with the average age being 70–80 years at the time of diagnosis [24]. In the aging process, senescent cells accumulate, and in some aspects, the signs of aging can resemble malignancies. In addition to intrinsic cellular changes, other fundamental aging processes, such as downregulation of the immune system, are also responsible for tumorigenesis. As we age, our immune system becomes less resilient and less efficient in detecting and fighting cancer cells [26]. This change is known as immunosenescence. Hematopoiesis, thymic involution, and the development, maturation, migration, and homeostasis of peripheral lymphocytes are all altered by the immunosenescence state. As a result, aging impacts how the acquired immune system functions. It does this by altering the surface of T-cell receptors, reducing the quantity of naive T-cells, altering their distribution, decreasing B lymphocytes, and building up aged T-cells, which are then accompanied by an increase in proinflammatory cytokines. The susceptibility of older people to infections is greatly impacted by these changes [27]. There are several reports of malignancies in the genitalia caused by LR-HPV [24, 28, 29], one of which manifest as vulvar SCC in geriatric patient [24].

SCC of the vulva has many clinical variations but generally may present as a white or erythematous tumor with a papillomatous surface. In some cases, erythematous macules, plaques and ulcerative lesions may be seen [3, 13, 14]. These skin lesions may be accompanied by pruritus and pain at the site of the lesion, but in some cases, they may not cause any subjective symptoms [1, 2, 13]. Symptoms that appear in more advanced stages include bleeding, discharge, lymph node swelling, pain when urinating, and difficulty defecating [1, 16]. The patient in the case report had a yellowish-white plaque lesion with a papillomatous surface that was sometimes pruritic and painful, especially when rubbed. There were no findings of advanced-stage vulvar SCC based on the patient's history and physical examination.

Histopathological examination is required to diagnose vulvar SCC [16]. Histopathological features of vulvar SCC include invasion of squamous epithelial cells from the epidermis into the dermal layer [20]. The tumor cells in vulvar SCC have large, hyperchromatic, polymorphic nuclei and eosinophilic cytoplasm [2, 30]. In addition, central keratinization and horn pearl formation may be observed [25]. In this case report, the histopathological findings were consistent with the features of vulvar SCC, including hyperplastic stratified squamous epithelium, parakeratosis, acanthosis, and some nuclei showing koilocytosis. The epithelial cells had transformed into a tumor mass consisting of polygonal round cells with tumor cell invasion into the underlying connective tissue stroma, which are composed of round, oval cells growing hyperplastically, densely packed, and clustered. The nuclei were polymorphic and hyperchromatic.

Buschke–Löwenstein tumor (BLT), or giant condyloma acuminatum, is a rare, slow-growing tumor of the anogenital region, which also strongly associated with low-risk HPV types 6 and 11 [31, 32]. Clinically, it presents as a large, cauliflower-like mass in the genital or perianal area, often exceeding 10 cm, arising from long-standing condyloma acuminatum [31]. Histologically, it is characterized by papillomatosis, hyperkeratosis, parakeratosis, acanthosis, and koilocytosis. Diagnosis relies on clinical presentation, patient history, and histopathological evaluation [31, 32]. Despite its benign histological appearance, BLT demonstrates locally aggressive behavior, with malignant transformation in up to 56% of the cases [31]. This case can be considered as BLT based on the lesion's gradual progression, positive HPV types 6 and 11, and histopathological findings such as koilocytosis, acanthosis, and parakeratosis. However, the relatively small lesion size (3.6 cm), absence of the typical exophytic, cauliflower-like morphology, and definitive histopathological evidence of malignant transformation—characterized by tumor cell invasion and nuclear pleomorphism—support the diagnosis of well-differentiated SCC of the vulva. Thus, the diagnosis of BLT can be excluded in favor of SCC.

Vulvar SCC lesions are considered invasive when tumor cells have invaded through the basement membrane into the underlying stroma [1, 3]. According to the World Health Organization, the prognosis of vulvar SCC is more strongly influenced by factors such as the HPV status, depth of stromal invasion, and lymph node involvement rather than histologic grade alone [33]. In this case, although the patient was histologically diagnosed with a well-differentiated vulvar SCC, further staging evaluation was performed by the Gynecologic Oncology division, including pelvic ultrasound, Pap smear, and bilateral inguinal FNAB. The assessment revealed no evidence of lymph node involvement or distant metastasis. Although rare, HPV-dependent SCC can be caused by low-risk HPV types 6 and 11, with clinical manifestations resembling AGW. The risk of malignancy development in low-type HPV must be considered, especially in elderly.

## Data Availability

The data that support the findings of this study are available on request from the corresponding author. The data are not publicly available due to privacy or ethical restrictions.
